# The therapeutic effect of ultrasound targeted destruction of schisandrin A contrast microbubbles on liver cancer and its mechanism

**DOI:** 10.2478/raon-2024-0019

**Published:** 2024-03-07

**Authors:** Xiaohui Wang, Feng Wang, Pengfei Dong, Lin Zhou

**Affiliations:** Department of Interventional Therapy, First Affiliated Hospital of Dalian Medical University, Dalian Liaoning, China; Department of Ultrasound, The First Affiliated Hospital of Zhengzhou University, Zhengzhou University, Zhengzhou, Henan, China; Department of Traditional Chinese Medicine, the Second Affiliated Hospital of Zhengzhou University, Zhengzhou, Henan, China; Department of Pharmacology, The First Affiliated Hospital of Zhengzhou University, Zhengzhou University, Zhengzhou, Henan, China

**Keywords:** ultrasonic targeted destruction, schisandrin A contrast microbubbles, liver cancer, mechanism of action

## Abstract

**Background:**

The aim of the study was to explore the therapeutic effect of ultrasound targeted destruction of schisandrin A contrast microbubbles on liver cancer and its related mechanism.

**Materials and methods:**

The Span-PEG microbubbles loaded with schisandrin A were prepared using Span60, NaCl, PEG-1500, and schisandrin A. The loading rate of schisandrin A in Span-PEG composite microbubbles was determined by ultraviolet spectrophotometry method. The Walker-256 cell survival rate of schisandrin A was determined by 3-(4,5)-dimethylthiahiazo (-z-y1)-3,5-di-phenytetrazoliumromide (MTT) assay. The content of schisandrin A in the cells was determined by high performance liquid chromatography. Ultrasound imaging was used to evaluate the therapeutic effect *in situ*. Enzyme linked immunosorbent assay (ELISA) was used to measure the content of inflammatory factors in serum. Hematoxylin-eosin (HE) staining was used to observe the pathological changes of experimental animals in each group. Immunohistochemistry was used to detect the expression of hypoxia inducible factor-1α (HIF-1α), vascular endothlial growth factor (VEGF) and vascular endothelial growth factor receptor 2 (VEGFR-2) in tumor tissues, and western blot was used to detect the protein expression of phosphoinositide 3-kinase (PI3K)/AKT/mammalian target of rapamycin (mTOR) signaling pathway in tumor tissues.

**Results:**

The composite microbubbles were uniform in size, and the particle size distribution was unimodal and stable, which met the requirements of ultrasound contrast agents. The loading rate of schisandrin A in Span-PEG microbubbles was 8.84 ± 0.14%, the encapsulation efficiency was 82.24±1.21%. The IC50 value of schisandrin A was 2.87 μg/mL. The drug + microbubbles + ultrasound (D+M+U) group had the most obvious inhibitory effect on Walker-256 cancer cells, the highest intracellular drug concentration, the largest reduction in tumor volume, the most obvious reduction in serum inflammatory factors, and the most obvious improvement in pathological results. The results of immunohistochemistry showed that HIF-1α, VEGF and VEGFR-2 protein decreased most significantly in D+M+U group (*P* < 0.01). WB results showed that D+M+U group inhibited the PI3K/AKT/mTOR signaling pathway most significantly (*P* < 0.01).

**Conclusions:**

Schisandrin A had an anti-tumor effect, and its mechanism might be related to the inhibition of the PI3K/AKT/mTOR signaling pathway. The schisandrin A microbubbles could promote the intake of schisandrin A in tumor cells after being destroyed at the site of tumor under ultrasound irradiation, thus playing the best anti-tumor effect.

## Introduction

Ultrasound contrast agent microbubbles were microparticles used to enhance the contrast of ultrasound images.^1^ The latest generation of ultrasound contrast agents had started to use microbubbles to carry drugs or genes, which could improve the effect of ultrasound imaging and achieve the purpose of treating diseases.^2^

Liver cancer is a common malignant tumor of the digestive system and the second most common human malignant tumor after lung cancer.^3^ At present, although the research on liver cancer had made a great breakthrough, it was still an important risk factor that seriously affected the life and quality of life of patients. At present, the treatment of liver cancer was mainly based on the stage and the age of the patient. The commonly used treatment methods include surgery, radiotherapy, chemotherapy, vaccines, and so on.^4^ In the chemotherapy of liver cancer, cisplatin-based combination chemotherapy was commonly used in clinical practice.^5^ Liver tumors were highly sensitive to platinum drugs and had good curative effect. However, the side effects and drug resistance of platinum drugs were also important factors affecting the application of platinum drugs.^6^ Despite the continuous improvement of surgery, radiotherapy and technology, and the emergence of chemotherapy drugs, the survival time and quality of patients had not been fundamentally improved.

Ultrasound-targeted microbubble destruction (UTMD) was a technology that improved the efficacy of targeted drugs by increasing the absorption of targeted drugs into cells.^7^ This technology mainly used microbubbles to localize “explosive” ultrasound irradiation and released the drugs they carried.^8^ At the same time, the shock caused by ultrasound and microbubble rupture increased the local cell permeability, generates reversible sonopores, and promotes drug entry into the nucleus, which could improve the efficiency of drug intervention in tumor cells.^9^ Secondly, the protection of microbubbles could prevent the drug from being metabolized and degraded by the body, thereby reducing the bioavailability of the drug, so that it could reach the target organ or tissue directly through the blood circulation.^10^ Many preclinical studies and few clinical studies reported the use of microbubble-assisted ultrasound for the delivery of wide range of therapeutics into primary liver tumors or liver mets.^11^ Schisandrin A is a bioactive lignan isolated from the traditional Chinese medicine *Fructus schisandrae chinensis*. Studies showed that schisandrin A had many pharmacological effects, such as anticancer, hepatoprotection, antiinflammation, which was worthy of further research and development in the future.^12^ Our previous study found that Schisandrisin A significantly reduced the inflammation level of HepG2 cells; improved the oxidative stress state; downregulated transforming growth factor beta 1 (TGF-β1), vascular endothlial growth factor (VEGF), phosphoinositide 3-kinase (PI3K), and Akt mRNA levels; inhibited the expression of the PI3K-Akt signaling pathway, and had a significant anti-tumor effect on tumor cells with high activity and small molecular weight, which was an ideal candidate for the production of contrast-enhanced ultrasound microbubbles.^13^ Therefore, in this study, Schisandin A was loaded into Span-PEG microbubbles to make ultrasound contrast agent and to play an anti-tumor effect on the lesions of liver cancer, which was rarely reported. This study would provide a new reference for the treatment of liver cancer.

## Materials and methods

### Instrumentation

Ultrasonic cell crushing instrument (Ningbo Xinzhi Biological Technology Co., LTD.), Doppler ultrasound diagnostic instrument (Kunshan Ultrasonic Instrument Co., LTD.), Zeta potential/particle size instrument (British Malvern Instrument Co., LTD.), SW-CJ-1D single side vertical air supply purification table (Suzhou Zhijing Purification Equipment Co., LTD., Jiangsu Province), HZQA Constant temperature incubator (Jintan Shenglan Instrument Manufacturing Co., LTD.), LX-C50L vertical automatic electric heating pressure steam sterilizer (Beijing Sibo Shengda Technology Co., LTD.), scanning electron microscope (Japan Electronics Co., LTD.), Ultrasound imaging instrument (mindray M9cv, Superficial probe), Bio-Rad 680 iMark Microplate reader (American Bio-Rad Co., LTD.)

### Reagents

Span60 (Tianjin BASF Chemical Co., LTD.), PEG1500 (Tianjin Guangfu Fine Chemical Research Institute), NaCl (Tianjin Beilian Fine Chemical Development Co., LTD.), schisandrin A (Chengdu Manst Biotechnology Co., LTD. HPLC ≧ 98%), phenol and sulfuric acid (Tianjin Kemio Chemical Reagent Co., LTD.), Walker-256 (number: 399-88-2) was obtained from Shanghai Hongshun Biotechnology Co., LTD. MEM (containing NEAA) basal medium (Procell PM150410) and fetal bovine serum (Procell 164210) was obtained from Pricella Biotechnology Co., LTD. Fixative solution (4% Paraformaldehyde, P1110) was obtained from Shanghai solarbio Bioscience & TechnologyCo., LTD. Enzyme linked immunosorbent assay (ELISA) kit tumor necrosis factor-α (TNF-α) (ab236712), interleukin-1β (IL-1β) (ab255730) and interleukin-6 (IL-6) (ab234570) was obtained from abcam Bioscience & TechnologyCo., LTD. Hematoxylin-eosin (HE) Stain Kit (G1120) was obtained from Solarbio Bioscience & TechnologyCo., LTD. Immunohistochemistry kit hypoxia inducible factor-1α (HIF-1α) (IHC0103715), VEGF(IHC0100011) and vascular endothelial growth factor receptor 2 (VEGFR-2) (IHC0102817) was obtained from Shanghai CaiYOU industrial Co., LTD. WB kit: Primary antibody p-PI3K (bs-6417R), PI3K(20584-1-AP), p-Akt (bs-0876R) was obtained from Bioss Co., LTD, AKT (60203-2-Ig), p-mammalian target of rapamycin (mTOR) (67778-1-Ig), mTOR (66888-1-Ig), was obtained from Proteintech Group, GAPDH was obtained from Hangzhou Hua ‘an Biotechnology Co. LTD, Secondary antibody (SA00001-1) was obtained from Bioss Co., LTD.

### Experimental cells

Walker-256 cell (Free of mycoplasma infection, cells were derived from ascites of liver cancer in rats) were cultured in RPMI 1640 medium containing 10% fetal bovine serum and incubated at 37°C in 5%CO_2_ incubator. The cells were routinely digested and subcultured with 2.5 g/L trypsin, and the logarithmic growth phase cells were used for experiments.

### Experimental animals

36 SD (Sprague Dawley, male, 6 weeks, 180−200 g, wide type rats) rats were obtained from Henan Laboratory Animal Center. Animal experiment ethics was approved by the Ethics Committee of the First Affiliated Hospital of Zhengzhou University (No. KY2023-006).

### Preparation and analytical characterization of Span-PEG microbubbles loaded with schisandrin A

450 mg Span60, 900 mg NaCl, 450 mg PEG-1500 and 300 mg schisandrin A were weighed, placed in a mortar and thoroughly ground, dissolved in 40 mL PBS phosphate buffer solution, and heated to 80°C in a magnetic heating mixer, and stirred and dispersed evenly. Then, the solution was continuously sonicated at 570 W power for 6 min using an ultrasonic cell disruptor by acoustic cavitation method, while nitrogen gas was continuously injected into the above solution. A uniform milky yellow liquid mixture was prepared and centrifuged in an ultracentrifier at 2 000 g for 8 min. After centrifugation, a stratified solution was obtained. The upper and middle layers were removed and placed in a 250 mL separating funnel, washed with an equal volume of PBS phosphate buffer, and left to stand. The middle layer microbubbles were collected and freeze-dried to obtain Span-PEG ultrasound contrast agent microbubbles loaded with schisandrin A.^14^ The size and shape of the microbubbles were observed by scanning electron microscopy (SEM). Detailed operation details were as follows: A small amount of microbubble powder was coated to one side of the double-sided glue and the other side was fixed on the stage of the scanning electron microscope. The surface morphology of the drug microbubbles was observed and photographed by scanning electron microscopy under a high voltage of 15 kV at a magnification of 5000. The particle size distribution and Zeta potential of the microbubbles were determined by ZS90 laser particle size analyzer. Detailed operation steps were as follows: Added pure water into the sample tank as the dispersing agent, turned on the ultrasonic disperser and set the intensity to 7, turned on pump switch after 2 minutes, adjusted the pump speed to 2680 r/min, specified water as the dispersing agent in the TAB, and other parameters were determined, clicked “Start”, measured the sample, and saved the results. Then changed the measurement conditions and re-measured until the next measurement results were basically in line with the last measurement results, then the last measurement results are the particle size measurement results of the sample. This experiment was independently repeated three times with consistent results.

### Determination of loading rate of schisandrin A in Span-PEG composite microbubbles

The loading rate of schisandrin A in Span-PEG microbubbles was determined by ultraviolet spectrophotometric method. The standard of schisandrin A was prepared at concentrations of 2.5 μg·mL^−1^, 5 μg·mL^−1^, 10 μg·mL^−1^, 20 μg·mL^−1^, 30 μg·mL^−1^, 40 μg·mL^−1^ and 50 μg·mL^−1^, respectively. The absorbance value was measured at 254 nm, the standard curve was drawn, and the regression equation was calculated. Span-PEG microbubbles loaded with schisandrin A were weighed 10 mg, dispersed in distilled water for ultrasonic release for 2 h, filtered, and constant volume to a 10 mL volumetric flask. The absorption wavelength of schisandrin A in Span-PEG composite microbubbles at 254 nm was determined by the same method. The schisandrin A loading rate was calculated according to the standard curve. This experiment was independently repeated three times with consistent results.

### Determination of the cell survival rate of schisandandin A by 3-(4,5)-dimethylthiahiazo (-z-y1)-3,5-diphenytetrazoliumromide (MTT) assay

The standard Schisandandin A was diluted in double dilutions in cell culture medium, so that the final concentrations in cell culture medium were 64 μg/mL, 32 μg/mL, 16 μg/mL, 8 μg/mL, 4 μg/mL, 2 μg/mL, 1 μg/mL and 0 μg/mL, respectively. Walker-256 cells with logarithmic growth were seeded in 96-well plates and divided into eight groups. And culture medium containing the corresponding concentration of drug was added to each well (1×10^4^/mL), the cells and the drug were incubated in an incubator for 72 h. Then, 10 μL of freshly prepared 5 mg/mL MTT solution was added to each well and continue to culture for 4 hours. The supernatant was discarded and dissolved by adding 200 μL dimethylsulfoxide (DMSO), and the absorbance value was measured at 490 nm by microplate reader. The drug concentration in the control group was zero. In the blank group, only culture medium, MTT and DMSO were added. The cell survival rate = (Experimental group OD - blank group OD)/(Control group OD - blank group OD). The 50% inhibitory concentration (IC50) was calculated by IC50 software to define the concentration range of schisandrin A in this experiment.^16^ Fitting and calculation of cell survival curves were calculated with GraphPad Prism 9.0.

The above MTT assay was also used to detect following cell survival rate in control (C), microbubbles (M), ultrasound (U), drug (D), drug + ultrasound group (D+U), drug + microbubbles + ultrasound group (D+M+U) group. This experiment was independently repeated three times with consistent results.

### Anti-tumor cell experiment assessment in different groups

The cells in logarithmic growth phase were used for the experiment and randomly divided into 6 groups: simple microbubbles group (M): the cells were resuspended in cell culture medium containing 5% microbubbles; simple ultrasound group (U): the cells were exposed to 300 kHz, 0.25 W ultrasound for 10 s; simple drug group (D): the cells were resuspended in cell culture medium containing 2.5 μg/mL schisandrin A; Drug + ultrasound group (D+U): The cells were incubated with 2.5 μg/mL schisandin A and then exposed to 300 kHz, 0.25 W ultrasound for 10 s; Drug + microbubbles + ultrasound group (D+M+U): After routine digestion and centrifugation, the cells were added with cell culture medium containing 2.5 μg/mL Schisandandin A and 5% microbubbles, and then ultrasound irradiation was performed under the same conditions as the previous group. Control group (C): The digested and centrifuged cells were resuspended and cultured in conventional culture medium.^17^ Cell survival rate was measured by MTT assay. This experiment was independently repeated three times with consistent results.

### The intracellular content of schisandrin A for quantitative determination

Sample collection: The treated cells of each group were cultured in 24-well plates with 6 multiple wells in each group. After 24 hours of culture, the cells of each group were routinely digested, centrifuged and collected, rinsed 3 times with PBS, and the last time was fixed in 1 mL double distilled water. Cells were destroyed using an ultrasonic cell morcellator to obtain a double distilled aqueous solution of intracellular fluid in each group. The chromatographic conditions were as follows: Kro-masil C18(250 mm×4.6 mm, 5 μm) column; The mobile phase was methanol-0.1% glacial acetic acid aqueous solution (82:18). Flow rate :1mL/min; Detection wavelength: 254 nm; The column temperature was 30°C. The chromatogram of schisandrin A samples and intracellular liquid standard was drawn under the selected chromatographic conditions.^18^ This experiment was independently repeated three times with consistent results.

### Establishment and grouping of rat liver cancer model

After 7 days of adaptive feeding, all groups of animals were inoculated with Walker-256 cells in the liver under ultrasound guidance to establish an orthotopic liver cancer model. When the tumor gradually grew to 35mm^2^, the drug treatment was started. The rats were divided into 6 groups with 6 rats in each group: control group (injected with equal volume of normal saline), microbubble group (injected with microbubbles via the tail vein without ultrasound), ultrasound group (injected with equal volume of normal saline with ultrasound), ultrasound + schisandrin A group (injected with schisandrin A via the tail vein), ultrasound + microbubble group (injected with microbubbles via the tail vein), and ultrasound + schisandrin A microbubble group (injected with schisandrin A microbubble via the tail vein). The liver parts of all animals were subjected to low-frequency ultrasound treatment at 300 kHz, 2.0 W/cm^2^, PRF(Pulse Repetition Frequency) 1 kHz, DC(Duty Cycle) 20%, PNP(Peak Negative Pressure) 0.5 MPa, with 10 s irradiation, 10 s interval, and a total of 20 min. The injection dose of schisandrin A was 20 mg/kg, and the microbubble injection dose was 0.3 mL/kg. The treatment was given every 3 days for a total of 16 days. Tumor observation: Ultrasound was used to observe the tumor growth at the inoculation site 7 days and 16 days after treatment. This experiment was independently repeated three times with consistent results.

### The treatment evaluation effect *in situ* by ultrasound imaging

In this study, mindray M9cv Doppler ultrasound was used to collect the *in situ* tumor images of animals in each group before and after treatment. The acquisition method was as follows: under the guidance of ultrasound, the tumor site was determined, and the longest and shortest diameters of the tumor were measured at the same time. This experiment was independently repeated three times with consistent results.

### Serum inflammatory factors detection

Twenty-four hours after the last treatment, blood samples were collected from the abdominal aorta of rats and stored in a test tube without anticoagulant. The samples were placed at 37°C for coagulation, and after blood coagulation, the samples were equilibrated and centrifuged (4°C, 2000 g, centrifugal radius was 7.5 cm), and the final obtained supernatant was the serum. The contents of TNF-α, IL-1β and IL-6 in serum were determined by enzyme-linked immunosorbent assay. The specific operation steps are as follows: 10 μL standards and 10 μL samples were added into the wells of the corresponding reaction plates. 40 μL TNF-α/IL-1β/IL-6 Biotin and 40 μL TNF-α/IL-1β/IL-6 POD were added to each well. The plates were mixed gently for 30 seconds, the wells were sealed, and the plates were incubated at room temperature for 45 minutes. Washed the plate: dump all the liquid in the plate, washed the reaction plate with washing solution (add 350 μL of washing solution to each well), and removed water droplets (pat dry on thick absorbent paper): washed 5 times repeatedly. 100 μL of chromogenic solution was added to each well, gently mixed for 10 seconds, and incubated at room temperature for 20 minutes. Added 100 μL stop solution to each well. Gentle mixing for 30 s: OD values were read at 50 nm within 30 min. This experiment was independently repeated three times with consistent results.

### Histopathological examination

Fresh liver tumor tissues were taken and fixed with fixative solution for more than 24 hours. The tissues were removed from fixative solution and trimmed in a fhood with a scalpel. Then, the tissues were dehydrated and immersed in wax, embedded, sectioned, hematoxylin staining, eosin staining, dehydrated and sealed. Finally, microscopic examination and image acquisition and analysis were performed. Pathological evaluation was made by pathologists under a microscope. Sample preparation was performed as follows: sampling, fixation, dehydration, transparency, wax immersion, embedding, and sectioning. The procedure for HE staining was as follows: The sections were successively washed in xylene I 10 min, xylene II 10 min, absolute ethanol I 5 min, absolute ethanol II 5 min, 95% alcohol 5min, 90% alcohol 5 min, 80% alcohol 5 min, 70% alcohol 5 min, distilled water. The sections were stained with Harris hematoxylin for 3−8 min, washed with tap water, differentiated in 1% hydrochloric acid alcohol for 7 seconds, rinsed with tap water, returned to blue in 0.6% ammonia water, and rinsed with running water. Sections were stained in eosin staining solution for 1-3min. The slices were successively placed in 95% alcohol I 5, min, 95% alcohol II 5 min, absolute ethanol I 5 min, absolute ethanol II 5 min, xylene I 5 min, xylene II 5 min for dehydration and transparency. The slices were taken out of xylene to dry slightly and sealed with neutral gum. This experiment was independently repeated three times with consistent results.

### The expression of HIF-1α, VEGF and VEGFR-2 by immunohistochemistry

The paraffin sections of liver tumor tissue were deparaffinized to water, and then underwent antigen repair, endogenous peroxidase blocking, serum blocking, primary antibody, secondary antibody, DAB staining, nucleus counterstain, dehydration and sealing. Finally, the sections were taken out of xylene to dry slightly, and sealed with sealing glue for microscopic examination. Specific IHC procedures are as follows: Paraffin sections were routinely deparaffinized to water. 3% hydrogen peroxide was used for 10 min at room temperature to inactivate endogenous enzymes, and the cells were washed twice with distilled water. The sections were immersed in 0.01 mol/L citrate buffer solution (pH 6.0), heated to boiling in microwave oven, and then turned off. After an interval of 5 min, the sections were cooled repeatedly and washed twice × 3 min with PBS buffer (pH 7.2−7.6). The blocking solution was added droppers at room temperature for 20min. Toss off any excess liquid without washing. Appropriate primary antibodies (Rat Anti-VEGF 1:300; Rat Anti-VEGFR2 1:200; Rat Anti-HIF-1a 1:200), incubated at 37°C for 2 h. Washed 3 min x 3 times with PBS buffer (pH 7.2−7.6). The working solution of biotinylated secondary antibody was added at 20~37°C for 20min. Washed 3 min × 3 times with PBS buffer (pH 7.2−7.6). Working solution of horse-radish enzyme-labeled streptavidin was dropped and washed 3 min × 3 times with 20~37°C, 20 min, PBS buffer (pH 7.2−76). The reaction time was controlled under the DAB chromoscope at room temperature, generally between 5 and 30 min. Light counterstained with hematoxyli n, dehydrated, sealed with transparent neutral gum, and observed under microscopy. This experiment was independently repeated three times with consistent results.

### Protein expression levels detection of PI3K-AKT-mTOR signaling pathway

The tumor tissue was taken and detected by Western blottting. About 200 mg of fresh tumor tissue was weighed, then liquid nitrogen was added while grinding in a mortar, the ground tissue was weighed in a peeled and precooled centrifuge tube. Then, the tumor tissue was fully lysed with RIPA (Radio Immuno Precipitation Assay) lysate containing 50 mM Tris (pH 7.4), 150 mM NaCl, 1% Triton X-100, 1% sodium deoxycholate, 0.1% SDS, and inhibitors containing sodium orthovanadate, sodium fluoride, EDTA, leupeptin. The total protein was extracted and the protein content was determined by BCA method. The protein samples (50 μg) were denatured, subjected to 12% polyacrylamide gel electrophoresis, transferred to polyvinylidene fluoride (PVDF) films, and blocked with 5% skim milk for 2 h at 4°C. Then, antibodies against PI3K, p-AKT, AKT, p-AKT, mTOR and pmTOR proteins were added (diluted 1:1 000) and incubated at 4°C overnight. The membranes were washed with TBST buffer, the secondary antibody (dilution 1:10 000) was added, and the membranes were incubated for 2 hours at room temperature. The membranes were washed with TBST (Tris Buffered Saline Tween20) buffer, and after adding ECL (Electro Chemi Luminescence) solution, the membranes were exposed and developed in an automatic chemiluminescence gel imaging analysis system. This experiment was independently repeated three times with consistent results.

### Statistical analysis

SPSS 22.0 statistical software was used for all statistical analyses of this study. The measurement data were expressed as mean ± standard deviation. Comparison between groups was performed using analysis of variance. *P* < 0.05 for the difference was considered statistically significant.

## Results

### Span-PEG microbubbles loaded with schisandrin A exhibited a stable physical and chemical properties

**FIGURE 1.**
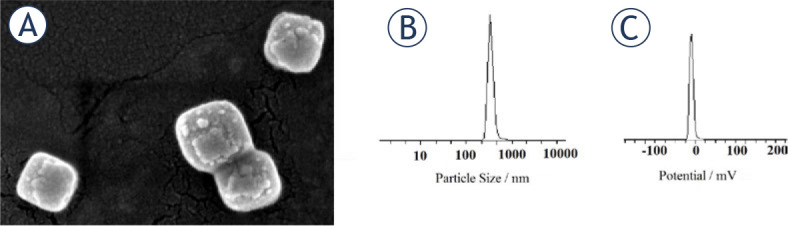
Analytical characterization of Span and polyethylene glycol (Span-PEG) microbubbles loaded with schisandrin A **(A)** The structure SEM of schisandrin A microbubbles; **(B)** The particle size distribution; **(C)** Zeta potential of schisandrin A microbubbles, (n = 6).

Figure 1A shows the structure SEM of Span-PEG composite microbubbles loaded with schisandrin A. The prepared microbubbles have smooth surface and uniform particle size. Figure 1B and 1C show the particle size distribution and Zeta potential of Span-PEG composite microbubbles, respectively. From the measurement results and distribution curves, it can be seen that the composite microbubbles have uniform size and single peak particle size distribution. The average particle size is 595.3 nm, less than 700 nm, and the Zeta potential is −18.8 mV, which is relatively stable. It meets the requirements as an ultrasound contrast agent.

### Span-PEG composite microbubbles showed a satisfactory loading rate of schisandrin A

According to the experimental method, the standard curve of schisandrin A was drawn. The standard curve equation of schisandrin A was: Y = 0.0121X-0.0012, *R*^2^ = 0.9992.

The absorbance value of schisandrin A in Span-PEG composite microbubbles was measured and substituted into the standard curve to calculate the loading rate of schisandrin A. The results are shown in Table 1. The loading rate of schisandrin A in Span-PEG composite microbubbles was 8.84 ± 0.14%, the encapsulation efficiency was 82.24 ± 1.21%.

**TABLE 1.** Loading rate of schisandrin A in Span and polyethylene glycol (Span-PEG) composite microbubbles

**Number of experiments**	**Absorbance value**	**Rate of load/%**
1	0.524	8.68
2	0.536	8.88
3	0.541	8.96
Mean	0.534	8.84
SD	0.009	0.14

### Schisandrin A could reduce cell survival rate

**FIGURE 2.**
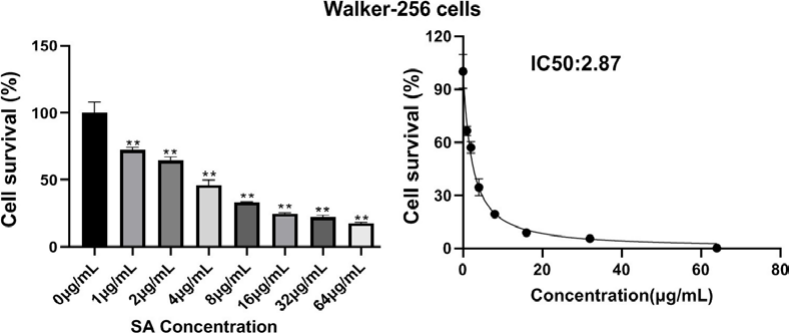
The Walker-256 cell survival rate of schisandrin A (n = 6; compared with control group [0 μg/mL], ^**^P < 0.01).

The results of MTT assay showed that the cell survival was inhibited to varying degrees after treatment with different concentrations of schisandrin A for 24h. The cell survival rates of 0 μg/mL, 1 μg/mL, 2 μg/mL, 4 μg/mL, 8 μg/mL, 16 μg/mL, 32 μg/mL, 64 μg/mL schisandrin A on Walker-256 cells after 24 hours were 100.00%, 72.36%, 64.52%, 45.90%, 33.39%, 24.78%, 21.97%, 17.44%, respectively. The IC50 value was 2.87 μg/mL. The concentration of schisandrin A selected for this experiment was 2.5μg/mL, as shown in Figure 2.

### U+M+D treatment could effectively inhibit cell survival

**FIGURE 3.**
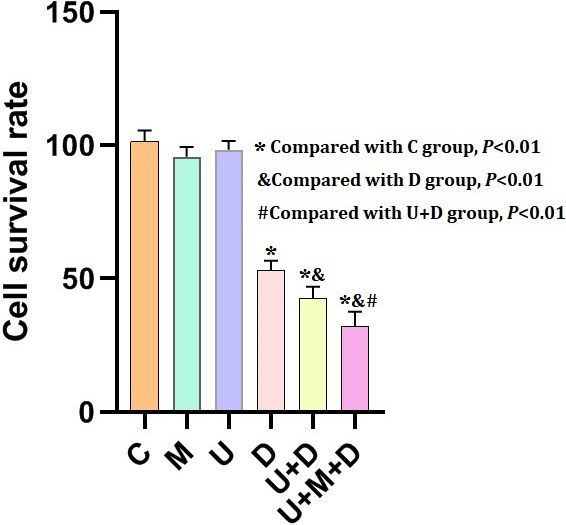
Anti-tumor cell experiment assessment in different groups (n = 6; ^*^Compared with C group, P < 0.01; &Compared with D group, P < 0.01; #Compared with U+D group, P < 0.01)

Compared with the control group, the cell survival rate of each experimental group decreased significantly: group C (100%) > group U (98.31%) > group M (95.68%) > group D (53.14%) > group U+D (42.53%) > group U+M+D (32.17%). There were significant differences among the groups (F = 626.5, P < 0.0001), group D *vs.* group D+U (MD = −0.32587, P < 0.001), group D *vs.* group D+M+U (MD = −0.52608, P < 0.001), group D+U *vs.* group D+M+U (MD = −0.32673, P < 0.001), as shown in Figure 3.

### U+M+D treatment could significantly increase the intracellular content of schisandrin A

**FIGURE 4.**
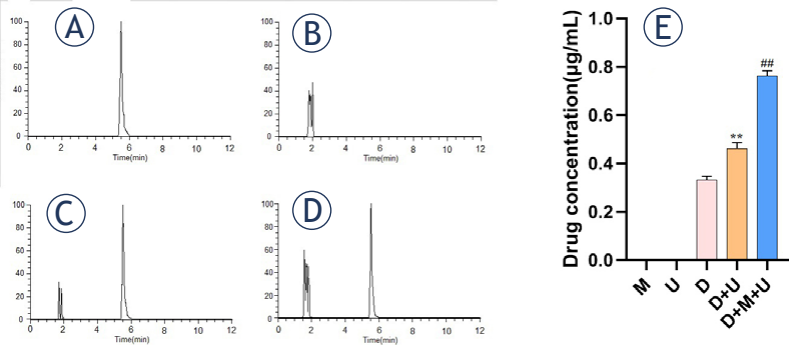
The content of schisandrin A in the cells of each group (A. Chromatogram of standard schisandrin A; B. Chromatogram of blank intracellular fluid; C. Chromatogram of blank intracellular solution + standard schisandrin A; D. Chromatogram of the tested intracellular liquid; E. Comparison of drug concentrations between different groups, n = 6; ^**^Compared with C group *P* < 0.001; ##Compared with D group *P* < 0.001)

Under the selected chromatographic conditions, the chromatographic profiles of schisandrin A standard, blank intracellular liquid, blank intracellular liquid + schisandrin A standard and the tested intracellular liquid were obtained (Figure 4A−D). The standard curve of schisandrin A was drawn to obtain the linear regression equation Y = 0.0544X+0.0128 (*R*^2^ = 0.9997), and the linear range was 0−6.4 μg/mL. The average concentration of schisandrin A in each group was calculated by taking the peak area of schisandrin A into the equation: group M (0 μg/mL); group U (0 μg/mL); Group D 0.33 μg/mL; Group D+U 0.46 μg/mL; Group D+M+U 0.76 μg/mL. There were significant differences in intracellular drug concentrations among the groups (F = 587.5, *P* < 0.0001), Group D *vs.* Group D+U (MD = −0.13667, *P* < 0.001), Group D *vs.* Group D+M+U (MD = −0.43500, *P* < 0.001), Group D+U *vs.* Group D+M+U (MD = −0.29833, *P* < 0.001), shown in Figure 4E.

### U+M+D treatment showed the best anti-tumor effect

**FIGURE 5.**
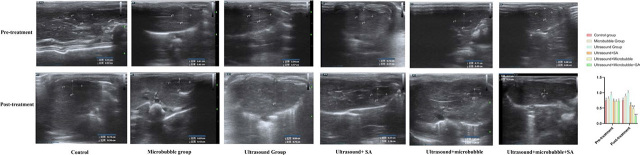
The changes in tumor size before and after drug treatment detected by Ultrasound images (n = 6; ^*^Compared with Pre-treatment group, *P* < 0.05; ^**^Compared with Pre-treatment group, *P* < 0.01).

The experimental results showed that the tumor in the control group (without treatment drugs), M (microbubbles) group and U (ultrasound) group had progressed and enlarged, and the other different treatment groups had significant effects before and after drug treatment (*P* < 0.05). Among them, the tumors in the ultrasound + schisandrin A group and the ultrasound + microbubble group showed a certain degree of atrophy (*P* < 0.05). The most significant effect was in the ultrasound + schisandrin + A microbubble group (*P* < 0.01). The results suggest that schisandrin A has a certain anti-tumor effect, and the microbubbles loaded schisandrin A can promote the intake of schisandrin A in tumor cells after blasting at the tumor site under ultrasound irradiation, thus playing the best anti-tumor effect, as shown in Figure 5.

### Serum inflammatory factors (TNF-α, IL-6, IL-1β) decreased obviously in U+M+D group

**FIGURE 6.**
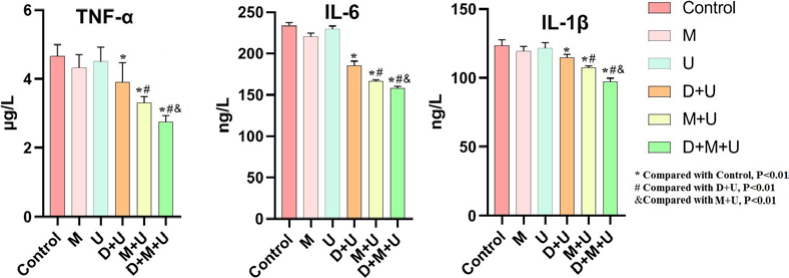
Detection of serum inflammatory factors (tumor necrosis factor-α [TNF-α], interleukin-6 [IL-6], interleukin-1β [IL-1β]) in different group (n = 6, ^*^Compared with C group, P < 0.01; #Compared with D+U group, P < 0.01; &Compared with M+U group, P < 0.01).

The experimental results showed that the levels of inflammatory factors in the control group (without therapeutic drugs) were higher, and there was no significant improvement for the levels of inflammatory factors in M group and U group. However, there were significant differences among the groups for TNF-α, (F = 73.698, *P* < 0.001), Group C *vs.* Group D+U (MD = 0.745, *P* < 0.01), Group C *vs.* Group M+U (MD = 1.228, *P* < 0.01), Group C *vs.* Group D+M+U (MD = 2.060, *P* < 0.01), Group D+U *vs.* Group M+U (MD = 0.483, *P* < 0.01), Group D+U *vs.* Group D+M+U (MD = 1.315, *P* < 0.01), Group M+U *vs.* Group D+M+U (MD = 0.831, *P* < 0.01). There were significant differences among the groups for IL-6, (F = 828.16, *P* < 0.001), Group C *vs.* Group D+U (MD = 47.280, *P* < 0.01), Group C *vs.* Group M+U (MD = 68.040, *P* < 0.01), Group C *vs.* Group D+M+U (MD = 74.818, *P* < 0.01), Group D+U *vs.* Group M+U (MD = 20.760, *P* < 0.01), Group D+U *vs.* Group D+M+U (MD = 27.539, *P* < 0.01), Group M+U *vs.* Group D+M+U (MD = 6.78, *P* < 0.01). There were significant differences among the groups for IL-1β, (F = 230.955, *P* < 0.001), Group C *vs.* Group D+U (MD = 9.99, *P* < 0.01), Group C *vs.* Group M+U (MD = 17.54, *P* < 0.01), Group C *vs.* Group D+M+U (MD = 27.14, *P* < 0.01), Group D+U *vs.* Group M+U (MD = 7.56, *P* < 0.01), Group D+U *vs.* Group D+M+U (MD = 17.15, *P* < 0.01), Group M+U *vs.* Group D+M+U (MD = 9.60, *P* < 0.01). Compared with the control group, the levels of inflammatory factors in the ultrasound + schisandrin A (U+D) group and the ultrasound + microbubble group (U+M) showed a downward trend. The level of decrease was most pronounced in the ultrasound + schisandrin A + microbubble (U+M+D) group, as shown in Figure 6.

### The pathological improvement was most obvious in U+M+D group

**FIGURE 7.**
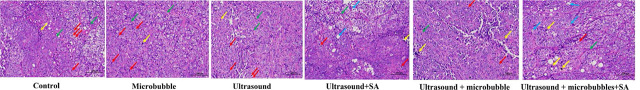
Histopathological changes in different groups (division of the tumor cell nucleus [red arrows], hyperplasia of collagen fibers [green arrows], necrosis of tumor cells [yellow arrows], and balloon-like swelling of tumor cells [blue arrows]).

In the control group, the tumor cells were closely arranged and the intercellular space was small, the nuclear showed atypia, the nuclei were large, the nuclear to cytoplasmic ratio was high, and mitotic figures were visible (red arrows), necrosis of tumor cells was occasionally observed (yellow arrow), the nuclei were pyknotic and hyperchromatic, there was a small amount of thin collagen fiber proliferation between the tumor cells (green arrow). There was no improvement in pathology for microbubble and ultrasound group. Compared with control group, in the ultrasound + schisandrin A group, the mitosis of tumor cell nuclei decreased (red arrows), there was a decrease in collagen fibers between tumor cells (green arrow), balloon-like swelling of tumor cells was observed (blue arrow). Compared with control group, in the ultrasound + microbubble group, tumor necrosis was increased (yellow arrow), the mitosis of tumor cell nuclei was decreased (red arrows), there was a decrease in collagen fibers between tumor cells (green arrow). Compared with control group, in the ultrasound + schisandrin A + microbubble group, tumor cell necrosis was significantly increased (yellow arrow), the mitosis of tumor cell nuclei was significantly decreased (red arrows), balloon-like swelling of tumor cells was significantly observed (blue arrow), there was a decrease in collagen fibers between tumor cells (green arrow), as shown in Figure 7.

### The immunohistochemical improvement was most obvious in U+M+D group

**FIGURE 8.**
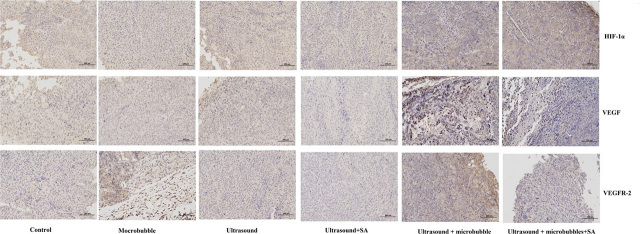
Immunohistochemical changes in different groups.

**FIGURE 9.**
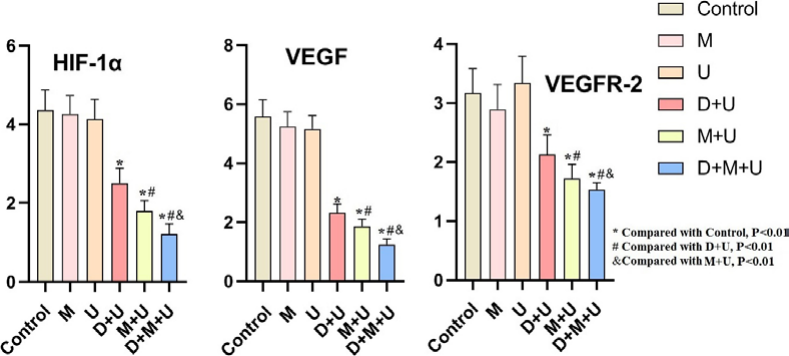
The expression of hypoxia inducible factor-1α (HIF-1α), vascular endothlial growth factor (VEGF) and vascular endothelial growth factor receptor 2 (VEGFR-2) proteins in the tumor tissues (n = 6, ^*^Compared with C group, P < 0.01; #Compared with D+U group, P < 0.01; &Compared with M+U group, P < 0.01)

The results of immunohistochemistry showed that the control group (without treatment drugs) had a large staining area and strong staining intensity. There was no improvement in immunohistochemical result for microbubble and ultrasound group. Compared with the control group, the staining area of ultrasound + schisandrin A group was smaller and the staining intensity was weakened. Compared with the ultrasound + schisandrin A group, the staining area of the ultrasound + microbubble group and the ultrasound + schisandrin A microbubble group gradually became smaller, as shown in Figure 8. The above results showed that the expression of HIF-1α, VEGF and VEGFR-2 proteins in the tumor tissues of the ultrasound + schisandrin A group, the ultrasound + microbubble group and the ultrasound + schisandrin A microbubble group showed a weakening trend (*P* < 0.01), as shown in Figure 9.

### PI3K/AKT/mTOR signaling pathway was most inhibited in U+M+D group

**FIGURE 10.**
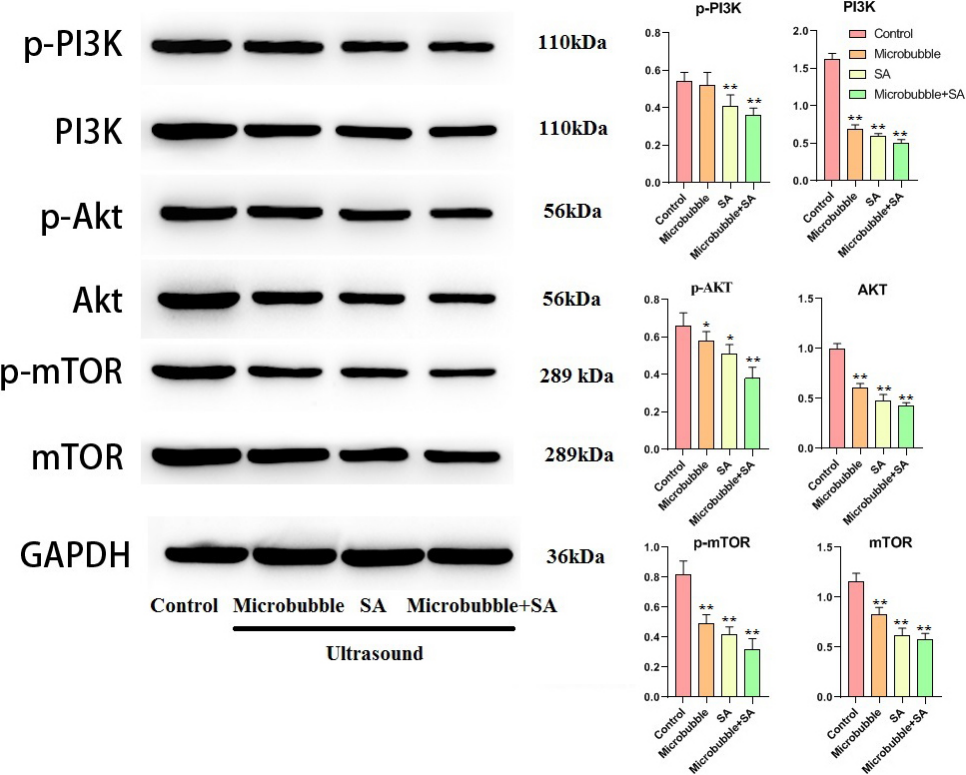
Protein expression results of phosphoinositide 3-kinase (PI3K)/AKT/mammalian target of rapamycin (mTOR) signaling pathway in tumor tissues (n = 6, ^*^Compared with Control, *P* < 0.05; ^**^Compared with Control, *P* < 0.01).

Compared with the control group, the relative protein expression levels of p-PI3K, PI3K, p-Akt, AKT, p-mTOR, mTOR proteins in the ultrasound + schisandrin A group and the ultrasound + schisandrin A microbubble group were significantly decreased (*P* < 0.05), and the ultrasound + schisandrin A microbubble group had the most obvious effect. It was suggested that schisandrin A can inhibit the PI3K/AKT/mTOR signaling pathway in tumor tissues. After ultrasonograph-assisted microbubble destruction, the uptake of schisandrin A by tumor cells was further promoted, so the tumor inhibition effect was more obvious, as shown in Figure 10.

## Discussion

Microbubbles (MBs) combined with ultrasound appeared to be an alternative therapeutic strategy for many diseases, and showed good clinical results.^19^ The combination of microbubbles and ultrasound had emerged as a promising method for local drug delivery. Microbubbles could be locally activated by a targeted ultrasound beam, which could result in several bio-effects, this was essential for targeted tumor therapy.^20^ In addition to the development of new types of ultrasound contrast agents, various imaging methods dedicated to contrast agents had been introduced, and some of them were now commercially available.^21^

This study found that the ultrasound irradiation microbubble contrast agent could increase the concentration of schisandrin A in Walker-256 cells, and the cytotoxic of schisandrin A on Walker-256 cells could be enhanced by the ultrasound irradiation microbubble contrast agent. The combination of ultrasonic microbubble contrast agent with low dose of cytotoxic drugs could achieve the killing effect of high dose of drugs on tumor cells when the drug was used alone, and it could reduce the toxic side effects on normal cells and tissues. It could not only improve the efficacy of chemotherapy, but also improve the tolerance of patients to chemotherapy.

On the basis of the cell experiment, this study carried out the verification experiment of the orthotopic rat liver cancer model. Imaging showed that the tumor volume of the D+M+U group was the smallest, and there was necrosis and liquefaction in the tumor. The pathological results of HE staining in each group showed that the tumor cells in the D+M+U group were loose, and a large number of necrotic cells appeared in the tissues. Although different degrees of cell necrosis and hemorrhage were observed in all groups except the control group, the changes in D+M+U group were more significant (*P* < 0.05). Studies had shown that HIF-1α, a transcription factor of VEGF, could regulate the expression of its downstream target gene VEGF through a variety of pathways.^22–23^ VEGFR-2 was the main receptor of VEGF for angiogenesis.^24^ The combination of VEGF and VEGFR-2 could promote the proliferation and extension of vascular endothelial cells and induce the formation of new blood vessels, which was conducive to the proliferation, invasion and metastasis of tumors. Therefore, HIF-1α/VEGF/VEGFR pathway was a key signal pathway to regulate tumor angiogenesis.^25^ In this study, immunohistochemical results showed that the expressions of HIF-1α, VEGF and VEGFR-2 in the D+M+U group were significantly decreased (*P* < 0.01), indicating that ultrasound combined with schisandrine A microbubbles could effectively inhibit the HIF-1α/VEGF/VEGFR pathway, thereby acting as an anti-tumor agent.

Inflammatory cytokines refer to the various cytokines involved in the inflammatory response. Among the many inflammatory cytokines, TNF-α, IL-1β, IL-6 and so on played a major role. In general, the content of cytokines in body was very low, and it participated in anti-inflammatory and anti-tumor effects with a variety of factors. Cytokines was highly expressed in liver cancer. After treatment, the drug and microbubble destroyed the microenvironment involved in tumor occurrence, inhibited the enzymes that promote tumor growth and proliferation, blocked the continued growth and metastasis of tumors, and then reduced the production of tumor necrosis factor in the body’s immune response, so that the serum level of tumor body after schisandrine A microbubbles treatment became low (*P* < 0.01).

PI3K/AKT/mTOR signaling pathway played an important role in the proliferation, metastasis, energy metabolism, autophagy and drug resistance of liver cancer.^26^ In recent years, the relationship between autophagy and tumors had attracted more and more attention from scholars. Studies had found that autophagy could inhibit tumor formation by reducing the accumulation of useless or damaged organelles and proteins, inhibiting oxidative stress and other processes. That was, promoting the autophagic activity of tumor cells and even inducing autophagic death could play an anti-tumor role. PI3K belonged to the lipid kinase family and could be activated by many cytokine receptors.^27^ The activation of PI3K led to the phosphorylation of Akt and further activates downstream signaling molecules such as mTOR, thereby inhibiting autophagy. mTOR was usually highly expressed in tumor cells.^28^ Inhibition of mTOR function and inactivation of PI3K/Akt/mTOR signaling pathway could induce autophagy.^29^ The results of western blot analysis in the present study showed that the p-PI3K, PI3K, p-Akt, Akt, p-mTOR, mTOR were significantly decreased in liver cancer tissues treated with schisandrin A combined with ultrasound (*P* < 0.01). These results suggested that schisandrin A combined with ultrasound might have an inhibitory effect on PI3K/Akt /mTOR signaling pathway in liver cancer.

In this study, we conducted a preliminary experiment on the selection of ultrasound parameters. For the intensity of ultrasound, when the intensity was lower than 1.5 W/cm^2^, the therapeutic effect was not good, and the microbubbles could not be fully burst to exert the anti-tumor effect. When the intensity was higher than 2.5 W/cm^2^, it would cause damage to the body’s own tissues, and the side effects would increase significantly. After repeated attempts, we found that 2.0 W/cm^2^ could reduce the toxic and side effects of drugs on normal tissues and organs, reduce the deposition of drugs in non-targeted sites, improve the tumor selectivity of drugs, and improve the tumor efficacy. Therefore, we had selected 2.0 W/cm^2^ as the optimal ultrasonic intensity parameters.

In addition, there were other ultrasound parameters that played a crucial role in the effect of treatment. PRF (Pulse Repetition Frequency) was a key parameter affecting blood flow effect. Higher PRF could significantly enhance the blood supply of the tumor area, produce stronger blood perfusion effect and obtain higher tissue drug concentration. The PNP (Peak Negative Pressure) of ultrasound was one of the parameters most related to the cavitation effect. The higher the peak negative pressure, the stronger the cavitation effect and the more serious the pathological changed. Therefore, it was necessary to choose moderate parameters to prevent damage to normal tissues. The ultrasonic cavitation effect occurred during the ultrasonic signal transmission. Therefore, increasing the duty cycle (DC) could prolong the actual ultrasonic signal transmission time, thereby enhancing the cavitation effect, accelerating the release of drugs and enhancing the therapeutic effect. We had systematically investigated the above parameters to ensure that the efficacy could be improved while the side effects could be reduced.

## Conclusions

In this study, the imaging microbubbles loaded with schisandrin A were prepared and their significant inhibitory effect on tumor cells was verified. The results showed that ultrasound combined with drug-loaded microbubbles could significantly increase the drug uptake in tumor cells, which was an effective way to improve the anti-tumor effect of drugs. The results in the orthotopic animal model of liver cancer showed that ultrasound combined with Schisandin A contrast microbubbles could significantly reduce the tumor volume, reduce the level of inflammatory factors in the animal body, and effectively inhibit the HIF-1α/VEGF/VEGFR pathway and PI3K-AKT-mTOR signaling pathway, which was a potential important therapeutic mechanism. This study would lay a scientific foundation for the improvement of the diagnosis and treatment of liver cancer.
